# Action of Alumina in Infantile Paralysis

**Published:** 1889-06

**Authors:** E. W. Berridge

**Affiliations:** London


### ACTION OF ALUMINA IN INFANTILE PARALYSIS.

E. W. Berridge, M. D., London.

When in the United States, in 1880, Dr. E. M. Hale related to me a remarkable case, which at my request he wrote out for me. The following is a copy of his statement. It will be seen that *Alumina* has cured a symptom supposed to belong exclusively to *Causticum:*

"A female child, three years of age, formerly healthy, was observed by the mother to *drag or swing the left leg.* When this had lasted several weeks I was called. On inquiry the following concomitant symptoms were elicited: *The child could only evacuate the bowels when standing,* and then only by *hard straining efforts, as if the abdominal muscles or the rectum were paralyzed.* When straining, the face became red, the eyes suffused, and the child trembled as from fear or pain.

"The nearest simillimum appeared to be *Alumina*, of which six pellets of the 30c was prescribed, to be given morning and night. In a week there was great improvement. The *Alumina* was then given only at night; and in another week the patient was cured, and has remained well to this date (three months).

"*Observations.*—The symptoms above given point unmistakably to an acute inflammation or congestion of the anterior portion of the spinal cord, or, according to Hammond, the 'anterior tract of gray matter.' It is reasonable to suppose that, had the disease not been averted in its early stage, atrophy and complete paralysis would have resulted. This case proves that *Alumina* has a specific action (1) on the lower portion of the cord; (2) on the anterior portion of the gray matter; and (3) that it will act promptly as a curative agent in the high potencies."

This is Dr. Hale's report. Doubtless his pathology is correct, but of what therapeutic utility is it? Will the knowledge that a remedy acts on the spinal cord enable any one to cure unless the totality of the symptoms agrees with the pathogenesis of the remedy? And if the totality harmonizes, what do we want with theories as to the minute lesions of the tissues? How will pathology differentiate between the action of *Alumina* and *Causticum* with regard to the peculiar features of the stool? So far as the scanty literature of this symptom shows, I think the *Causticum* symptom is not attended with severe straining, whereas this is an important feature of the *Alumina* symptom.

